# Second Toe Osteochondral Bone Graft for Resurfacing the Articular Surface of the Proximal Phalanx Head of the Thumb: A Case Report

**DOI:** 10.7759/cureus.55738

**Published:** 2024-03-07

**Authors:** Yaeesh Sardiwalla, Emmanuel O Olaonipekun, Pamela D Ball, Jouseph O Barkho

**Affiliations:** 1 Department of Plastic Surgery, McMaster University, Hamilton, CAN; 2 Department of Medicine, Royal College of Surgeons in Ireland, Dublin, IRL; 3 Department of Rehabilitation Science, McMaster University, Hamilton, CAN

**Keywords:** cartilage repair, trauma, arthroplasty, arthrodesis, thumb, autologous bone graft

## Abstract

The most widely accepted surgical management of a traumatized interphalangeal joint of the thumb is arthrodesis. However, in certain situations, specific functional and vocational demands require preserved movement at this joint. In the present case report, we describe harvesting the second toe proximal phalanx head as an osteochondral bone graft to recontour the proximal aspect of the thumb interphalangeal joint. The post-operative hand therapy regimen is described resulting in a pain-free functional range of motion. We conclude that when a motivated, healthy patient has specific functional goals, osteochondral bone grafting from the toe is a viable technique to maintain a functional range of motion.

## Introduction

The interphalangeal joint (IPJ) of the thumb allows flexion and extension of the distal phalanx, which guides precision movements, such as those of playing an instrument, building an electrical circuit, or wielding a firearm. Irrespective of age or occupation, this joint enhances one’s ability to participate in very specific vocational or occupational activities. When this joint is damaged, it can result in debilitating arthritic pain and loss of function. The most widely accepted treatment for a badly damaged thumb IPJ is arthrodesis [1]. Although arthrodesis is a reliable technique, it has a risk of non-union, infection, and compensatory changes at the adjacent metacarpal-phalangeal joint [2]. Furthermore, patients with psoriatic arthropathy are at an increased risk of complications following arthrodesis of the small joints of the hand [3]. Range of motion is permanently lost, which may cause impairment for patients who have specific needs.

When vocational or occupational demands require a mobile thumb IPJ after an injury disrupting the articular cartilage, the only solution is arthroplasty. In the present case study, we present a case of destruction of the articular surface of the head of the proximal phalanx of the thumb after a table saw accident. An autologous second-toe arthroplasty was successfully used to restore a gliding articular surface and motion and relieve pain.

## Case presentation

Informed consent was obtained from the patient to write this manuscript and use all photographs and a video. A 48-year-old, right-hand dominant, non-smoking police officer sustained a table saw injury to the dorsum of the right thumb while woodworking. The patient had no other comorbidities.

Radiographs showed a clear gouge in the articular surface of the head of the proximal phalanx and a preserved distal phalanx base. Evaluation in the emergency department revealed an open IPJ with a laceration of the terminal extensor pollicis longus (EPL) tendon. The wound was irrigated, and nonviable tissues were debrided. The tendon was repaired with a permanent monofilament suture and the thumb splinted in a gypsum plaster thumb spica. A gentle active motion was initiated three weeks post-injury.

**Figure 1:**
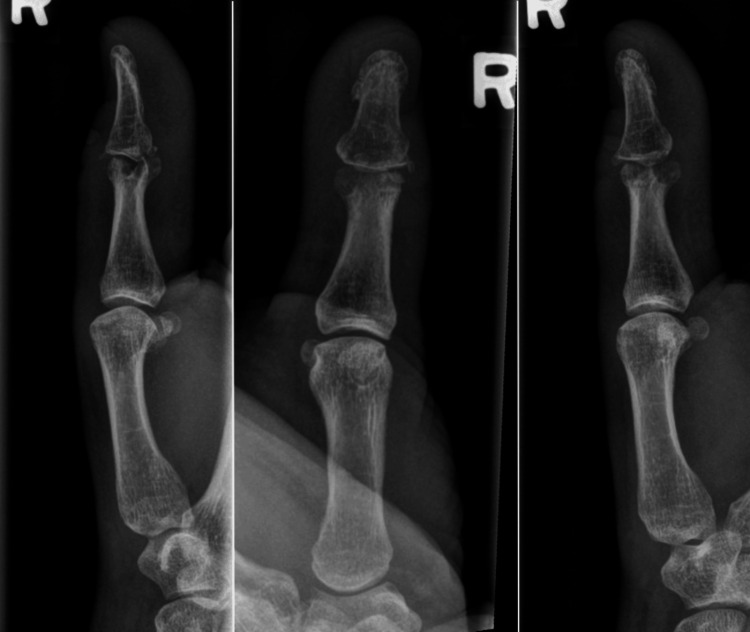
Lateral, anteroposterior, and oblique radiographs of the thumb demonstrating a gouge-like defect in the head of the proximal phalanx with a bony block to the distal phalanx base, preventing active flexion.

Evaluation at the two-month post-injury mark showed well-healed wounds with a thumb IPJ fixed in 180 degrees of extension and no active or passive range of motion. Attempted movement was painful. Repeat radiographs showed that the volar base of the distal phalanx was still entrapped within the gouged area of the proximal phalanx head (Figure 1). The patient was offered an arthrodesis to fix the thumb in a more functional position and resolve pain. The patient valued the motion of the IPJ, which was crucial to his occupation when gripping a firearm, and requested any modality that could restore motion.

Since the articular surface at the base of the distal phalanx was preserved and the geometry of the injury was that of a gouge in the coronal plane of the thumb, we reasoned that restoration of an articular gliding surface on the proximal phalanx head could theoretically restore pain-free motion. The proximal phalanx of the second toe bears a head with two condyles resembling that of the thumb proximal phalanx, although on a smaller scale. The patient was offered an autologous osteochondral bone graft of the head of the second toe proximal phalanx to the thumb, with a backup of arthrodesis should that fail. The case was planned with the hand occupational therapist who recommended a post-operative traction pin to allow static traction and off-loading the bone graft to minimize pressure necrosis on the bone graft.

Surgical technique

**Figure 2:**
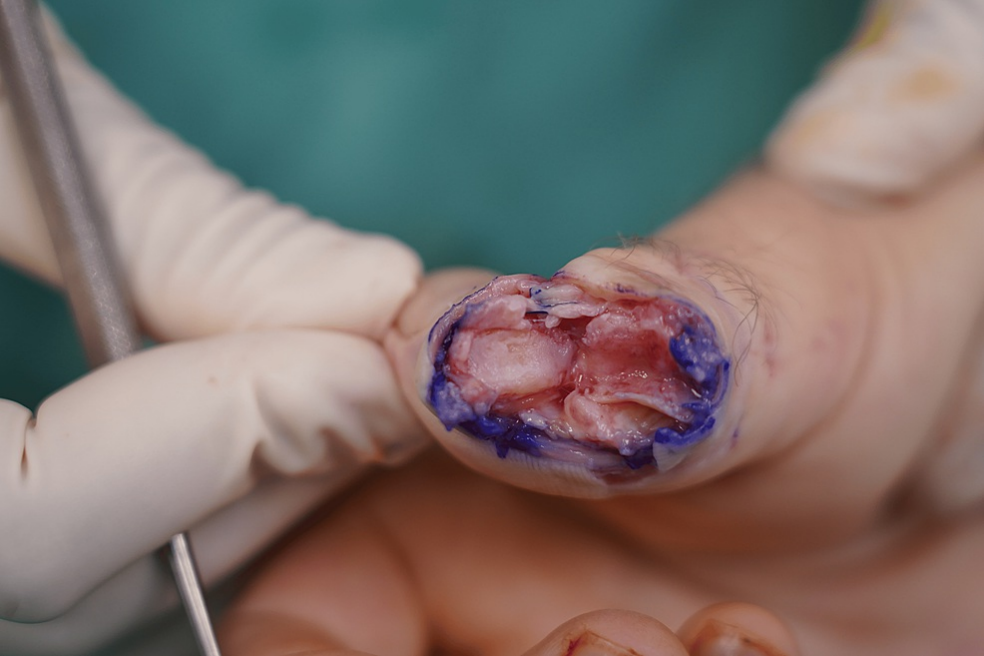
Intra-operative imaging of the bony defect in the head of the proximal phalanx caused by a table saw. The base of the distal phalanx shows abrasion of the cartilage but intact contour. A radial-based shotgun approach was used.

**Figure 3:**
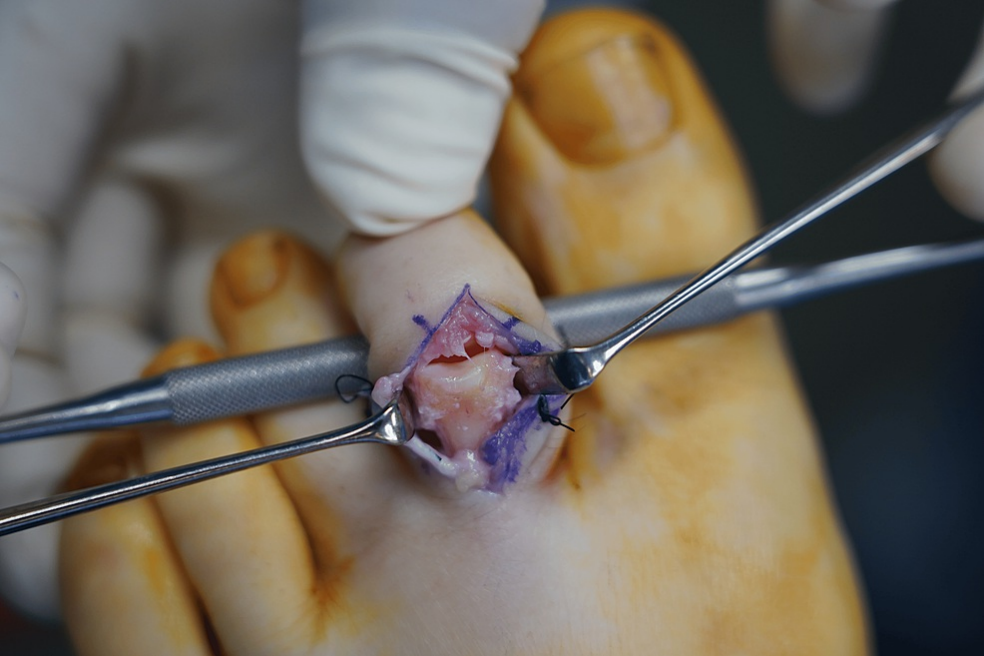
Intra-operative surgical exposure of the second toe proximal phalanx head.

**Figure 4:**
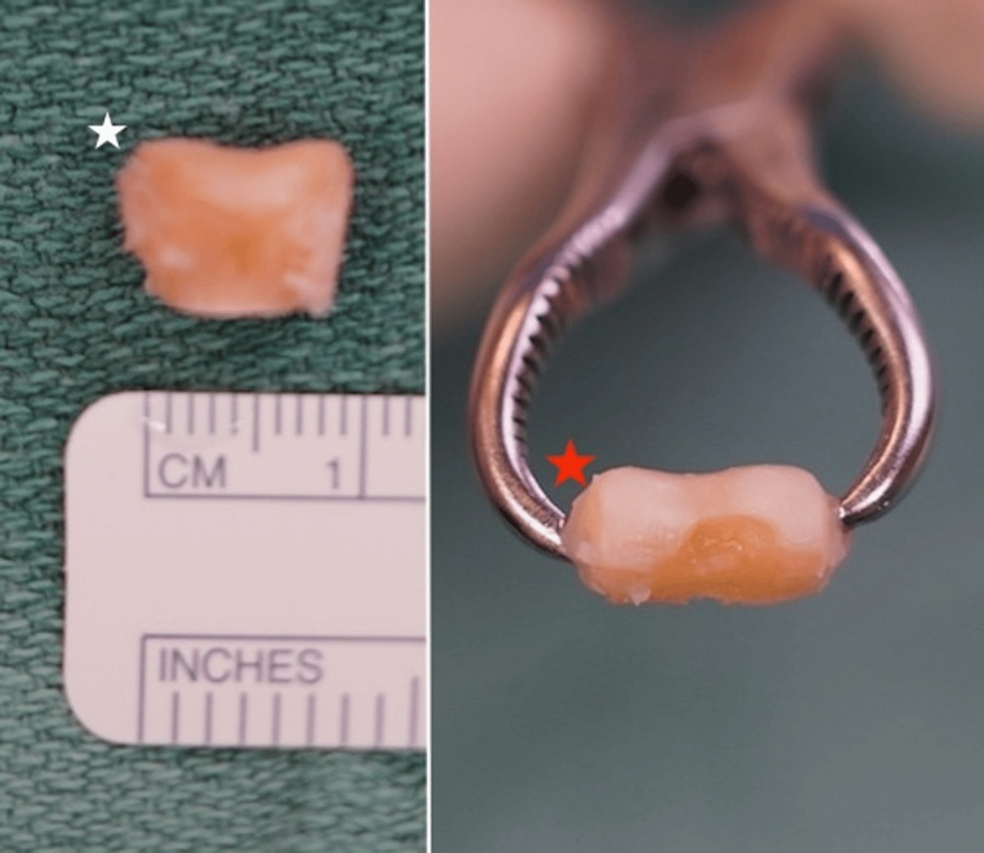
The second toe proximal phalanx head osteochondral graft. The white star indicates osteochondral graft before and the red star indicates graft after thinning and contouring with an electric burr.

**Figure 5:**
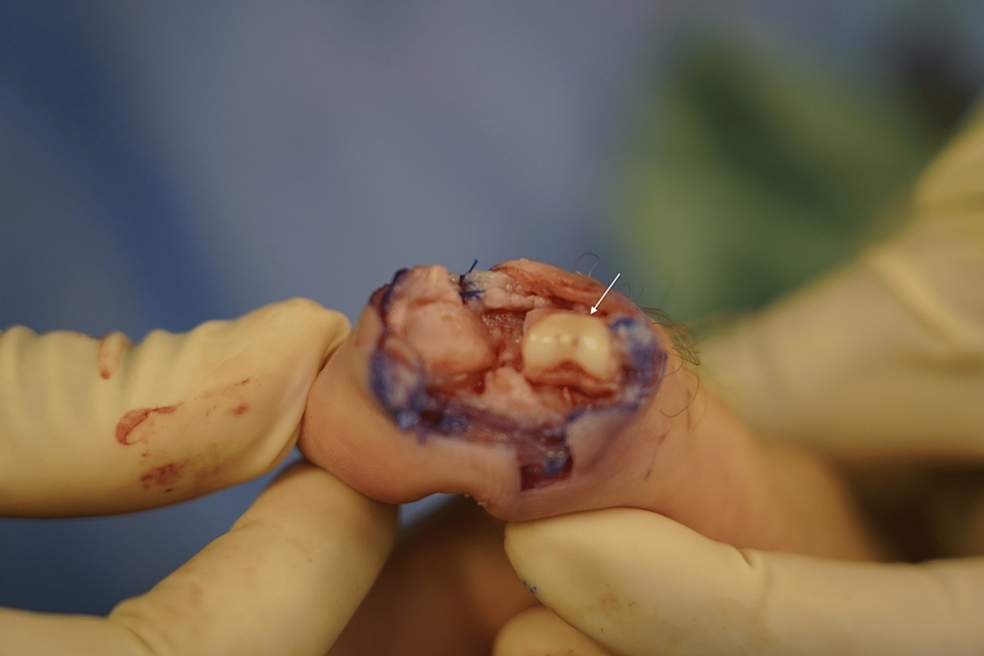
Intra-operative inset of the osteochondral bone graft. The white arrow indicates the inset of the bone graft into the bony defect of the thumb.

The patient was given prophylactic first-generation cephalosporin and general anesthetic with endotracheal intubation prior to incision. Under tourniquet control, the right thumb IPJ was approached from the radial side through a shotgun approach to avoid injury to the previously repaired EPL on the dorsal-ulnar side. This revealed the damage to the condyles of the proximal phalanx head (Figure 2). Under tourniquet control on the left lower limb, the second toe proximal phalanx head was approached from a dorsal curvilinear incision, midline splitting of the central slip, and transverse arthrotomy (Figure 3). The head of the proximal phalanx of the second toe was removed by osteotomy at the neck and then thinned and contoured with a round dental burr to maximize surface contact with the bony defect (Figure 4). The defect was also freshened to perfectly fit the graft, which was secured with two buried retrograde crossing K-wires (Figure 5). A traction pin was placed transversely through the base of the distal phalanx. The wounds were irrigated, and the thumb collateral ligaments and the volar plate were repaired with a permanent braided suture. At the second toe bony donor site, the base of the middle phalanx was resected to freshen the bone edge and the defect collapsed proximally. Two buried longitudinal K-wires were used for fixation to fuse the bones. The hand was protected with a gypsum plaster thumb spica with volar palm extension, and an Aircast-style boot for the left foot with immediate ambulation allowed.

Post-operative occupational hand therapy was initiated on post-operative day six. A hand-based static traction splint was fabricated to distract the distal phalanx base and prevent pressure necrosis on the bone graft. This was to be worn continuously except for hygiene and a gentle active range of motion three times a day.

**Figure 6:**
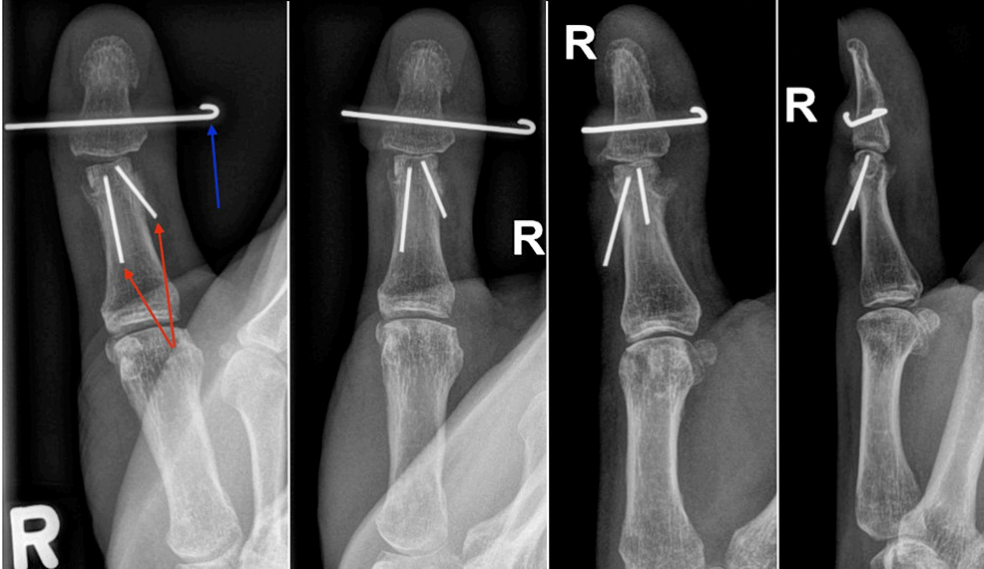
One-month post-operative radiographs showing two retrograde Kirschner wires securing the bone graft (red arrows) and transverse pin through the distal phalanx for static traction (blue arrow).

**Figure 7:**
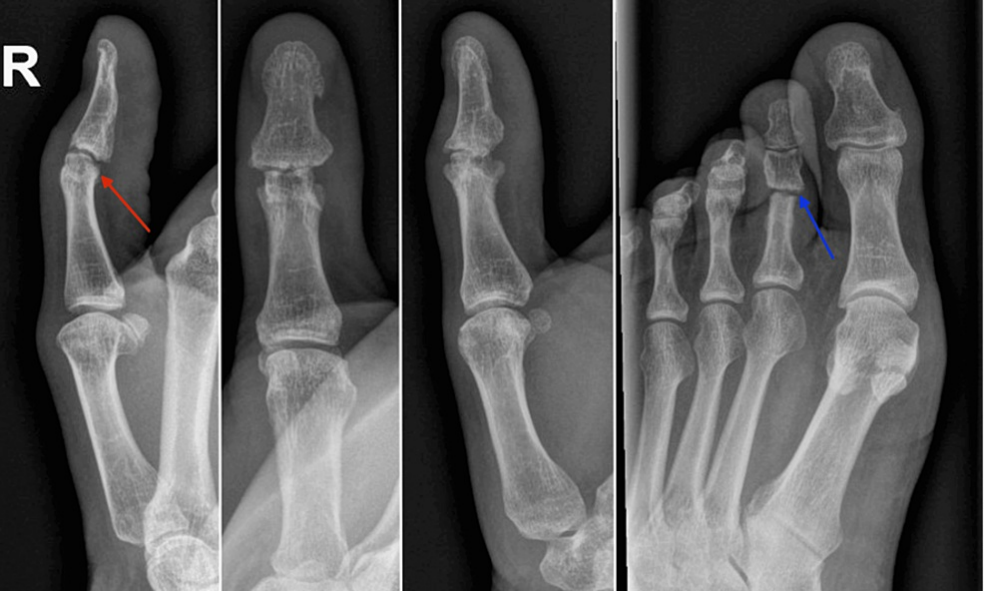
Three-month post-operative radiographs showing a well-healed osteochondral bone graft without resorption. On the lateral view, a small volar bony lip can be seen, which limits complete flexion (red arrow). The second toe donor site developed a pain-free fibrous union (blue arrow).

**Figure 8:**
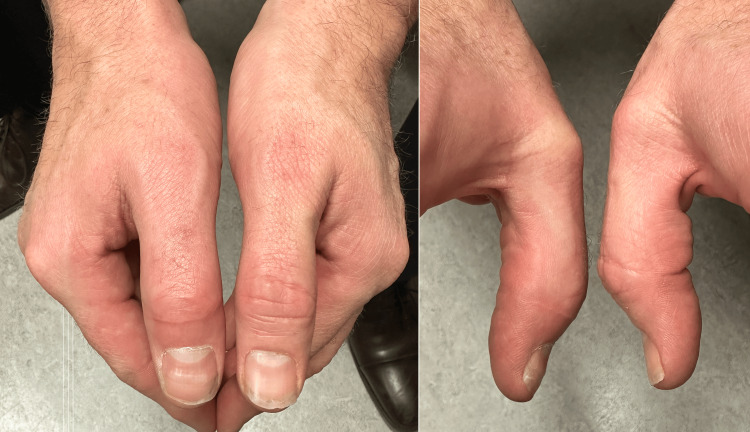
Cosmetic appearance of the right thumb post-operatively.

At the two-week mark post-operatively, a gentle passive range of motion was initiated. It was noted the thumb IPJ developed ulnar clinodactyly (passively correctible), likely due to attenuation of the radial collateral ligament secondary to the surgical exposure, despite repair. The splint was adjusted to deviate the distal phalanx radially, which corrected the angulation at the one-month mark (Figure 6). At the two-month post-operative mark, the K-wires from the thumb and toe were removed under local anesthetic, and full motion and force were permitted without the use of a splint. Therapy was targeted at restoring the spontaneous natural function of the thumb as well as grip and pinch training. At the three-month mark, the patient had right thumb IPJ flexion 47/58 degrees (active/passive, respectively), grip strength of 36 kg on the right, 38 kg on the left, lateral pinch of 5.25 kg on the right, and 7 kg on the left, and tip pinch of 2 kg on the right, and 4 kg on the left. The graft was well integrated although there was a bony lip from the native condyle edge volarly, which blocked further flexion (Figure 7). The range of motion is functional (Video 1), the patient reports pain-free use of the thumb, and the appearance is cosmetically acceptable (Figure 8). There were no problems with gripping a handgun.

**Video 1:** Post-operative active range of motion of the right thumb.

## Discussion

Autografts from the toe have been previously used to reconstruct the IPJs of the digits [4,5]. This report, however, represents the first case in the literature of second-toe autologous arthroplasty to successfully reconstruct the thumb IPJ. The condylar surface of the second toe proximal phalanx is analogous to that of the thumb proximal phalanx. It provides a gentle valley for which the articular prominence of the base of the distal phalanx can sit and glide. The size is smaller overall, limiting the use in severely damaged joints. This technique was possible because the articular surface of the distal phalanx was preserved and did not require reconstruction. While free tissue transfer of toe IPJs is described, this technique is complicated and requires microvascular expertise.

Arthroplasty of the thumb IPJ is not common. Only one other case report exists using a synthetic joint made of polyethylene, cobalt chrome, and titanium, and achieved excellent functional results [6]. The advantage of using autologous material over synthetic is that the tissues are revascularized. Therefore, the complications associated with synthetic materials are avoided, such as infection, extrusion, implant failure, hardware loosening, and peri-prosthetic fracture [7]. Furthermore, synthetic arthroplasties of the small joints of the hand usually require avoidance of heavy forceful use to avoid implant failure. The use of autologous tissue has its own pitfalls, including the creation of a donor site (with its own associated complications), failed integration, requirement for fixation techniques, and prolonged immobilization. However, autologous tissues may be more long-lasting than synthetic materials.

The thumb is most functional at a position of 18 degrees (±5 degrees) of flexion, and the functional arc of motion is 21 to 65 degrees (with an average of 40 degrees difference) [8]. The thumb, therefore, does not need as much range of motion as the metacarpal-phalangeal and proximal IPJs of the digits, which demand a functional range of 60 degrees on average [8]. Rather than mobility, the thumb demands pain-free stability to act as a post, which opposes the other digits during grasp. These properties make arthrodesis of the thumb joints favorable, except in specific situations that require motion, such as grasping a firearm in our case.

The osteochondral graft used in this case was primarily cancellous with a cartilaginous cap. The graft was thinned by precise burring, which provided maximal contact with the bony base and a shorter time to revascularize compared to larger pieces. A thinner bone graft is less likely to undergo internal necrosis. Cancellous grafts re-vascularize rapidly (two days to two weeks) due to the abundant lacunae and existing vascularity [9]. However, cancellous grafts are not structurally sound and may collapse under pressure during the immediate avascular period. This was the rationale for using static traction initially in our case for 35 days post-operatively.

## Conclusions

In conclusion, autologous graft arthroplasty of the IPJ of the thumb is a feasible solution in a very specific patient type. In our patient, the following factors led to the success of the operation: only one surface of the thumb IPJ required reconstruction, there was a specific task that required motion at the IPJ (gripping a firearm), the patient was a non-smoker with no comorbidities that challenged bone healing, and finally the patient was highly motivated to participate in the procedure and subsequent rehabilitation. When these factors are met, the patient and problem are optimized for success, and therefore joint motion can be preserved compared to an arthrodesis.
